# When RNA and protein degradation pathways meet

**DOI:** 10.3389/fpls.2014.00161

**Published:** 2014-04-23

**Authors:** Benoît Derrien, Pascal Genschik

**Affiliations:** ^1^Centre National de la Recherche Scientifique, Institut de Biologie Moléculaire des Plantes, Unité Propre de Recherche 2357, Conventionné avec l’Université de StrasbourgStrasbourg, France; ^2^Laboratoire de Biochimie et Physiologie Moléculaire des Plantes, Institut de Biologie Intégrative des Plantes ’Claude Grignon’, UMR CNRS/INRA/SupAgro/UM2, Montpellier CedexFrance

**Keywords:** RNA silencing, RISC, ubiquitin, autophagy, virus, proteasome

## Abstract

RNA silencing has become a major focus of molecular and biomedical research in the last decade. This mechanism, which is conserved in most eukaryotes, has been extensively studied and is associated to various pathways implicated in the regulation of development, in the control of transposition events, heterochromatin maintenance and also playing a role in defense against viruses. Despite of its importance, the regulation of the RNA silencing machinery itself remains still poorly explored. Recently several reports in both plants and metazoans revealed that key components of RNA silencing, such as RNA-induced silencing complex component ARGONAUTE proteins, but also the endonuclease Dicer are subjected to proteasomal and autophagic pathways. Here we will review these post-translational proteolytic regulations with a special emphasis on plant research and also discuss their functional relevance.

## A GLIMPSE IN THE RNA SILENCING PATHWAYS

RNA silencing involves processing of double stranded (dsRNA) by the enzyme Dicer, into small RNAs, 21–25 nucleotides in length called small interfering RNAs (siRNAs; reviewed in (Ghildiyal and Zamore, 2009; Voinnet, 2009; Krol et al., 2010). One of the two strands of each RNA fragment is then incorporated into a protein complex called RNA induced silencing complex (RISC) that invariably contains a member of the highly conserved ARGONAUTE protein (AGO) family. Once integrated into the RISC, siRNAs will base-pair to their target mRNA and induce their cleavage. The process of RNA interference (RNAi) is widely used for functional genomics and has also practical applications in therapeutics and agriculture. Most importantly, RNA silencing mediates resistance to exogenous pathogenic nucleic acids. Thus, important functions for small RNAs have emerged in the study of host-pathogen interactions and the most compelling illustration of the role of RNA silencing in defense is provided in the case of viral infections in plants, invertebrates and also more recently mammals, where populations of siRNAs are produced in infected cells directly by processing dsRNA molecules derived from the viral genome (Ding, 2010; Maillard et al., 2013). These viral-derived siRNAs are then incorporated into an antiviral RISC and turned back onto viral RNAs to trigger their degradation.

RNA silencing also regulates the expression of protein-coding genes. In this process, an important source of endogenous dsRNAs are primary transcripts of RNA-coding genes called pri-miRNAs which are processed, in the nucleus of metazoan cells, to 70-nucleotide stem-loop pre-miRNAs by the RNase III enzyme Drosha (Siomi and Siomi, 2010). After their export to the cytoplasm, pre-microRNAs are further processed via Dicer or Dicer-like (DCL) enzymes to produce miRNA duplexes. Plant genomes do not encode Drosha homologs, and all miRNA biogenesis steps at least in *Arabidopsis* are carried out by one of the four DCL proteins (Rogers and Chen, 2013). The microRNA (miRNA) duplex is separated, and one strand is selected as the 21-nucleotide mature miRNAs, whereas the other strand is degraded. Mature miRNAs are integrated into RISC complexes that repress the expression of one or more target mRNAs with complementary sequence by inhibiting mRNA translation or inducing their degradation. Thus miRNAs are predicted to regulate the expression of hundreds of mRNAs suggesting that they can regulate a significant proportion of the transcriptome (Leung and Sharp, 2010). Notably it has recently been shown that miRNAs are also subjected to turnover through degradation mechanisms implying both 3′–5′ and 5′–3′ exoribonucleases, adding another layer of complexity (Ramachandran and Chen, 2008; Chatterjee and Grosshans, 2009; Rüegger and Grosshans, 2012).

## REGULATION OF THE RNA SILENCING MACHINERY BY AUTOPHAGY

While the biogenesis and the function of small RNAs have been extensively studied in various biological processes across many organisms, less attention was paid on the regulation of the RNA silencing machinery itself. As indicated above, AGOs are core components of the RISC (Hutvágner and Simard, 2008; Vaucheret, 2008; Voinnet, 2009). These proteins have undergone a high degree of gene duplication in metazoans and plants, counting 8 and 10 genes in humans and *Arabidopsis*, respectively. Genetic and biochemical analyses revealed that *Arabidopsis* AGO1 plays a central role in both miRNA and si-mediated RNA silencing (Mi et al., 2008; Takeda et al., 2008). Based on its key role as effectors in RNA silencing, it is expected that AGO1 protein abundance must be strictly regulated, most likely at multiple levels. Hence, either an increase or a decrease in AGO1 protein content leads to significant effects on plant development (Vaucheret et al., 2004, 2006). The most studied and best-understood mechanism controlling AGO1 homeostasis is its negative regulation by miRNA168 (Vaucheret et al., 2006; Mallory and Vaucheret, 2009). In this pathway, the miRNA miR168 represses AGO1 transcript in an AGO1-dependant manner. Besides AGO1, other elements of the RNA silencing machinery, like DCL1 or AGO2 are also regulated via specific miRNAs, respectively, miR162 and miR403 (Xie et al., 2003; Allen et al., 2005). However, it became evident that AGO1 is also regulated at the post-translational level and in particular at the level of its stability.

The first evidence of selective AGO1 protein turnover was in the context of plant-viral interactions. *Arabidopsis* AGO1 is not only involved in the miRNA pathway, but together with AGO2 mediates antiviral defense (Alvarado and Scholthof, 2011). As a counter defense, viruses have elaborated various strategies to avoid silencing by expressing Viral Suppressors of RNA silencing (VSRs) proteins (Pumplin and Voinnet, 2013). Interestingly, it was found that certain VSRs, called P0 proteins from Poleroviruses, promote the degradation of AGO1 and thus presumably could impair RNA-based anti-viral immunity (Baumberger et al., 2007; Bortolamiol et al., 2007). This mechanism is conserved and was extended to VSRs of other viruses (Chiu et al., 2010; Fusaro et al., 2012). Interestingly, it was shown that P0 acts upstream of AGO1 loading and thus would prevent the formation of RISC (Csorba et al., 2010). This is supported by the fact that newly synthesized AGO1 after transient expression in tobacco leaves is subjected to P0-mediated destruction while endogenous AGO1 pre-assembled complex is P0-resistant. At the molecular level, viral P0 VSRs encode F-box proteins (Pazhouhandeh et al., 2006) that hijack the host SKP1-Cullin1-F-box protein (SCF) ubiquitin-protein ligase (E3) to promote ubiquitylation, which serves as a signal for degradation. This post-translational modification (PTM) regulates a broad range of physiologically and developmentally controlled processes in all eukaryotes (Ciechanover et al., 2000; Smalle and Vierstra, 2004). Because ubiquitylation of target proteins by SCF-type complexes most often leads to their proteasomal degradation, it was a surprise to find that the degradation of AGO1 by P0 was insensitive to inhibition of the proteasome (Baumberger et al., 2007). The mystery of AGO1 degradation pathway by the SCF^P0^ E3 ligase was, however, solved when it was reported that this process is mediated by autophagy (Derrien et al., 2012). Although recent studies already indicate a function of ubiquitylation in autophagy (McEwan and Dikic, 2011), this finding was nevertheless intriguing with respect to the presumed high selectivity of the P0-mediated ubiquitylation process, as degradation by autophagy is generally believed to be unspecific, even taking into account “selective autophagy” destroying protein aggregates and organelles.

**FIGURE 1:**
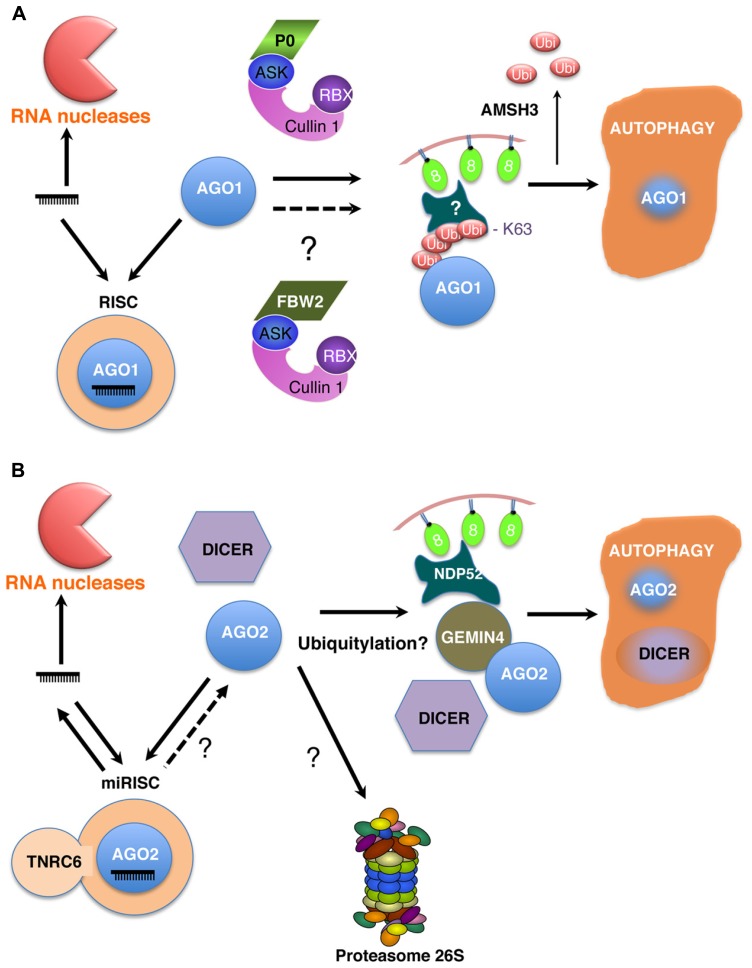
**Models for the turnover of AGO proteins in *Arabidopsis* (A) and mammalian cells (B).** Different levels of regulation operate on the homeostasis of RISCs. First, the steady-state levels of microRNAs are regulated by degradation processes involving different ribonucleases (RNases) acting either 3′–5′ or 5′–3′. Thus microRNAs most likely are in competition for AGO binding. Recent evidences essentially from metazoans indicate that at least some microRNAs can be released from RISCs, explaining their short half-lives. However, not only microRNAs but also AGO proteins are degraded. Thus in both plants and animal cells, it is now clearly established that AGO proteins are degraded by autophagy in an RNA free form prior RISC assembly. This mechanism also co-degrades other components of the silencing machinery such as DICER in mammals **(B)**. In *Arabidopsis*, the *polerovirus* protein P0 assembles an SCF^P0^ ubiquitin ligase to ubiquitylate AGO1 or an AGO1 associated protein **(A)**. Viral P0-mediated AGO1 degradation by autophagy also requires the deubiquitylating enzyme AMSH3. However, the identity of endogenous ubiquitin ligases involved in this process have not yet been unambiguously identified. The role of ubiquitylation in the turnover of human AGO2 is at present unclear but requires GEMIN4 and NDP52 **(B)**. Finally, whether upon guide RNA dissociation AGO2 would become accessible to autophagy and the role of the proteasome in AGOs degradation are other still open questions.

Because viruses usually hijack host cell machineries, it was conceivable that AGO1 protein turnover by autophagy may also occur in a P0-independent context. Hence, this prediction was confirmed when it was shown that mutations affecting miRNA biogenesis and/or accumulation and thus disturbing RISC assembly, also result in AGO1 degradation by autophagy (Derrien et al., 2012). This finding, however, raises the question of which is the endogenous ubiquitin-protein ligase (E3) that promotes ubiquitylation of AGO1 in a non-viral context. Notably *Arabidopsis* genome encodes several classes of E3s that are the key factors defining substrate specificity and among them more than 700 hundred F-box proteins (Vierstra, 2009). One good candidate to fulfill such a function is the *Arabidopsis* F-box protein FBW2 (Earley et al., 2010). FBW2 was identified by a genetic suppressor screen of a null allele of SQUINT (SQN), encoding a Cyclophilin-40 chaperon, a positive regulator of AGO1 activity. While FBW2 loss-of-function mutants do not exhibit an increase in AGO1 protein level, most likely because of the miR168-dependent feedback mechanism regulating AGO1 expression (Vaucheret et al., 2006), FBW2 overexpression significantly reduces AGO1 protein content (Earley et al., 2010). Interestingly, the proteasome inhibitor MG132 was also unable to block the FBW2-mediated degradation of AGO1, a situation reminiscent to the viral SCF^P0^ complex. At present it remains unclear whether FBW2 mediates AGO1 destruction by autophagy similarly to viral P0 (**Figure 1**), with which FBW2 does not share any significant sequence similarity beside an F-box motif.

Notably AGO1 is not the only *Arabidopdsis* AGO so tightly regulated at the post-translational level. Thus, at least in transient expression assays in tobacco leaves, P0 is also able to mediate the degradation of AGO2, AGO4-6, and AGO9 (Baumberger et al., 2007). Whether those AGOs are targeted by the endogenous SCF^FBW2^ is presently unknown, thought some of them have already been identified as ubiquitylated by proteomic approaches (Maor et al., 2007; Kim et al., 2013).

Is autophagy-mediated regulation of AGO proteins specific to the green linage? The answer is no and several findings suggest that the fate of the animal RNA silencing machinery shares some striking similarities with plants. Previous studies already reported that in mammals, AGO proteins are regulated at the post-translational level. For instance, in human cells, AGO2 (the only mammalian AGO producing RNA cleavage) is both hydroxylated and phosphorylated (Qi et al., 2008; Zeng et al., 2008). In particular hydroxylation was shown to influence both AGO2 subcellular localisation and stability, although the biological significance of this modification is still unclear.

Significant molecular insights on the post-translational control of metazoan AGO proteins emerged only recently. First it has been shown that the molecular chaperone HSP90 is required for the stability of mammalian AGO1 and AGO2 (Johnston et al., 2010). Thus inhibition of HSP90 function by geldanamycin triggered the degradation of both AGOs, an effect that could be alleviated, at least partially, by the proteasome inhibitor MG132. Interestingly, HSP90 does not bind AGO2 complexes that contain miRNAs and was therefore proposed to act upstream of RISC action indicating already that it is RNA-free AGO2 that is degraded in this pathway (Johnston et al., 2010). However, the first ubiquitin E3 ligase proposed to control mammalian AGO2 stability was the mouse Trim domain containing protein mLIN41 (Rybak et al., 2009). This protein is preferentially expressed in several stem cell niches and participates in the control of stem cell maintenance. mLIN41 physically interacts with AGO2 through its coiled-coil domain and promotes AGO2 ubiquitylation *in vitro* and *in vivo* through its RING and B-Box domains, all located in the Trim domain. Moreover, the ectopic overexpression of mLin41 reduced the level of endogenous Ago2 in embryonic carcinoma cells and this effect was attenuated by inhibition of the proteasome with MG132. However, more recent studies put into question the control of AGO2 stability by mLIN41 (Chang et al., 2012; Chen et al., 2012). In particular, it was shown that mLin41 promotes neuronal progenitor cell maintenance through FGF signaling by ubiquitylation of Shc SH2-binding protein 1 (SHCBP1), but not via the regulation of AGO2 stability (Chen et al., 2012).

While the turnover of AGO2 by the mLIN41-proteasome pathway will need further investigations, the degradation of Argonautes proteins by the autophagy pathway turned out to be conserved across kingdoms (**Figure 1**). Hence it was shown that both DICER and AGO2 levels increased in HeLa cells treated with chemical inhibitors known to block autophagy and in siRNA-depleted cells for different component of the autophagy pathway, such as ATG5, ATG6, ATG7 or NDP52 (Gibbings et al., 2012). Of particular interest was NDP52, a known autophagy receptor, which confers some cargo selectivity typically by recognizing conjugated ubiquitin (Rogov et al., 2014). At present the mechanism by how NDP52 recognizes AGO2 and DICER is unclear, but GEMIN4, a component of the multi-protein SMN (survival of motor neuron) complex, is required in this process eventually by interacting with both NDP52 and AGO2. Whether AGO2 ubiquitylation is a prerequisite to be directed to autophagy is unknown. In contrast DICER might be recruited by a mechanism independent of GEMIN4. Moreover similar to plant AGO1 decay (Csorba et al., 2010), mammalian AGO2 autophagy-mediated degradation occurs upstream of the formation of miRISC (Gibbings et al., 2012).

This novel paradigm of the post-translational control of the RNA silencing machinery exhibits nevertheless some variations. Hence in *Caenorhabditis elegans*, the Ago homologs ALG-1 and ALG-2 accumulate into aggregates in autophagy mutants only under certain stress conditions and the role of selective autophagy in their regulation under normal physiological conditions is presently unclear (Zhang and Zhang, 2013). Instead, AIN-1, a homolog of mammalian GW182/TNRC6 that interacts with AGO and mediates silencing, is clearly degraded by autophagy (Zhang and Zhang, 2013). AIN-1 colocalizes with SQST-1, the homolog of mammalian p62 that acts as a receptor for autophagic degradation of ubiquitylated protein aggregates and also directly interacts with Atg8/LC3 contributing to cargo specificity. This mechanism seems also to involve EPG-7 a scaffold protein linking cargo-receptor complexes with the autophagic assembly machinery (Lin et al., 2013). The putative role of ubiquitylation in the mechanism of AIN-1 destruction will nevertheless need further investigations.

## SOME PERSPECTIVES

It is clear that more work is required to better understand post-translational regulations of AGOs in both plants and metazoans. Moreover in plants, it will also be important to characterize the different protein complexes containing AGOs and their subcellular locations. We already know that plant AGO1 is present in both low and high molecular protein complexes that co-fractionate with small RNAs (Baumberger and Baulcombe, 2005; Qi et al., 2005; Csorba et al., 2010). Whether these multi-protein complexes resemble those identified in mammals (Filipowicz et al., 2008) remains, however, to be established. Moreover, evidence of two distinct cellular pools of AGO1 (siRNA versus miRNA loaded AGO1) RISCs was also recently established (Schott et al., 2012). In addition, in *Arabidopsis*, at least a fraction of AGO1 is also associated to membranes and isoprenoid biosynthesis which is important for membrane protein localization and trafficking, is required for miRNA function (Brodersen et al., 2012). Mammalian AGO2 was already known to bind to cellular membranes, most likely as a component of RISC (Cikaluk et al., 1999). AGO proteins are therefore present in cells as various pools representing likely different functional states. How are these different AGO protein pools regulated at the post-translational level and what is the impact of these regulations on RNAi function are major questions that will have to be solved.

Concerning the process of AGOs degradation by autophagy, an important issue will be to clarify the role of ubiquitylation. In plants, immunoprecipitation assays revealed an enrichment of polyubiquitin conjugates of AGO1 and/or an AGO1-associated protein and MLN-4924, a drug that inhibits the activity of cullin-RING ubiquitin ligases, impaired P0-dependant AGO1 degradation in *Arabidopsis* (Derrien et al., 2012). In mammals, AGO2 or one of its associated protein was also found ubiquitylated in cells treated with siRNAs to deplete autophagy activity (Gibbings et al., 2012). Notably, ubiquitin contains seven internal lysine residues and all can serve as conjugation sites to build up poly-ubiquitin chains that depending on their topologies can direct the substrate to the 26S proteasome or to the autophagy pathway (Grabbe et al., 2011; McEwan and Dikic, 2011). Therefore future experiments should reveal the identity of endogenous ubiquitin E3 ligases involved in this process, where and how they recognize AGOs or other associated proteins, the topology of the polyubiquitin chains that are generated and how these chains will be selected by the autophagy pathway.

Notably, at present we cannot rule out the possibility that the 26S proteasome also plays important functions in controlling the homeostasis of the RNA silencing machinery, as both proteolytic pathways may coexist, eventually in different cell types or specific developmental contexts. For instance, several studies incriminate the proteasome in controlling the stability of *Drosophila* and mammalian AGO effector proteins (Johnston et al., 2010; Smibert et al., 2013). Also in plants, the silencing suppressor protein P25 of *Potato virus X* (PVX) triggers AGO1 destabilization by the proteasome (Chiu et al., 2010). The mechanism by which this is achieved is unknown, but P25 might recruit a still unknown endogenous ubiquitin ligase complex to achieve such a function. Moreover, the Double-stranded RNA Binding protein (DRB4) that interacts with DCL4, one of the four Dicer-like proteins present in *Arabidopsis*, is also degraded by the proteasome after being recognized by the APC/C (anaphase promoting complex or cyclosome), a master ubiquitin protein ligase that usually targets cell cycle regulatory proteins (Marrocco et al., 2012). Thus to understand the contribution of proteasomal degradation versus the autophagy pathway in fine-tuning components of the RNA silencing machinery needs further investigations, both in metazoans and plants.

Finally, the most interesting question is what could be the physiological function(s) of these proteolytic pathways? The current model indicates that the stability of AGO proteins depends on miRNA biogenesis and thus unloaded AGOs are unstable (Derrien et al., 2012; Martinez and Gregory, 2013; Smibert et al., 2013). If AGO proteins are degraded essentially prior RISC assembly (Csorba et al., 2010; Johnston et al., 2010; Gibbings et al., 2012), the key regulatory step would be at the level of small RNA production that would compete for binding of available AGOs. In such a scenario, the P0 proteins from poleroviruses would destroy AGO1 at an early step to prevent viral siRNAs produced during infection to be incorporated into novel RISCs and this would compromise antiviral RNA silencing. However, what is the fate of AGO proteins once part of small RNA programmed RISCs? In mammals the half-life of Ago2 bound to small RNAs seems rather stable, at least under normal grow conditions (Johnston et al., 2010). Similarly, a half-life of 2–3 days of AGO1 RISCs was estimated in plants (Csorba et al., 2010). However, recent findings revealed that target RNAs could destabilize the interaction between human Ago2 and their corresponding guide RNAs, indicating that at least some RISCs can be unloaded (De et al., 2013). Such a dynamic loading and unloading mechanism might not only allow reprogramming of Ago2 by novel guide RNAs, but might also expose the protein to cellular degradation machineries such as the autophagy pathway. If this holds true, what would be the functional relevance of this degradation on RISC homeostasis and reprogramming?

## Conflict of Interest Statement

The authors declare that the research was conducted in the absence of any commercial or financial relationships that could be construed as a potential conflict of interest.
